# Typology of birth centres in the Netherlands using the Rainbow model of integrated care: results of the Dutch Birth Centre Study

**DOI:** 10.1186/s12913-017-2350-9

**Published:** 2017-06-21

**Authors:** Inge C. Boesveld, Marc A. Bruijnzeels, Marit Hitzert, Marieke A. A. Hermus, Karin M. van der Pal-de Bruin, M. Elske van den Akker-van Marle, Eric A. P. Steegers, Arie Franx, Raymond G. de Vries, Therese A. Wiegers

**Affiliations:** 1 Jan van Es Institute, Netherlands Expert Centre Integrated Primary Care, Wisselweg 33, 1314 CB Almere, the Netherlands; 2 Department of Obstetrics and Gynaecology, Erasmus University Medical Centre, PO Box 2014, 3000 CA Rotterdam, the Netherlands; 3 Department of Child Health, TNO, PO Box 2215, 2301 CE Leiden, the Netherlands; 4 Department of Obstetrics, Leiden University Medical Centre, PO Box 9600, 2300 RC Leiden, the Netherlands; 5 Midwifery Practice Trivia, Werkmansbeemd 2, 4907 EW Oosterhout, the Netherlands; 6 Department of Medical Decision Making, Leiden University Medical Centre, PO Box 9600, 2300 RC Leiden, the Netherlands; 7 Division Woman and Baby, University Medical Centre Utrecht, PO Box 85500, 3508 GA Utrecht, the Netherlands; 8 Academie Verloskunde Maastricht/Zuyd University, CAPHRI School for Public Health and Primary Care, PO Box 616, 6200 MD Maastricht, the Netherlands; 9 NIVEL (Netherlands Institute for Health Services Research), PO Box 1568, 3500 BN Utrecht, the Netherlands

**Keywords:** Birth centres, Integrated care, Collaboration, Classification, Typology

## Abstract

**Background:**

The goal of integrated care is to offer a continuum of care that crosses the boundaries of public health, primary, secondary, and tertiary care. Integrated care is increasingly promoted for people with complex needs and has also recently been promoted in maternity care systems to improve the quality of care. Especially when located near an obstetric unit, birth centres are considered to be ideal settings for the realization of integrated care. At present, however, we know very little about the degree of integration in these centres and we do not know if increased levels of integration improve the quality of the care delivered. The Dutch Birth Centre Study is designed to evaluate birth centres and their contribution to the Dutch maternity care system. The aim of this particular sub-study is to classify birth centres in clusters with similar characteristics based on integration profiles, to support the evaluation of birth centre care.

**Methods:**

This study is based on the Rainbow Model of Integrated Care. We used a survey followed by qualitative interviews in 23 birth centres in the Netherlands to determine which integration profiles can be distinguished and to describe their discriminating characteristics. Cluster analysis was used to classify the birth centres.

**Results:**

Birth centres were classified into three clusters: 1)“Mono-disciplinary-oriented birth centres” (*n* = 10): which are mainly owned by primary care organizations and established as physical facilities to provide an alternative birthplace for low risk births; 2) “Multi-disciplinary-oriented birth centres” (*n* = 6): which are mainly multi-disciplinary oriented and can be regarded as facilities to give birth, with a focus on integrated birth care; 3) “Mixed Cluster of birth centres” (*n* = 7): which have a range of organizational forms that differentiate them from centres in the other clusters.

**Conclusion:**

We identified a recognizable classification, with similar characteristics between birth centres in the clusters. The results of this study can be used to relate integration profiles of birth centres to quality of care, costs, and perinatal outcomes. This assessment makes it possible to develop recommendations with regard to the type and degree of integration of Dutch birth centres in the future.

**Electronic supplementary material:**

The online version of this article (doi:10.1186/s12913-017-2350-9) contains supplementary material, which is available to authorized users.

## Background

The essence of integrated care is a continuum of care for service users, crossing the boundaries of public health, primary, secondary, and tertiary care [1–3]. Integrated care is increasingly promoted for people with complex needs (e.g. multiple chronic diseases) and has more recently been recommended for maternity care systems [3]. Delivering integrated (birth) care demands both inter-professional and inter-organizational collaboration and therefore requires development of new clinical practices [4]. Birth centres, especially when they are located near an obstetric unit, are considered to be ideal settings for integrated care [5] and are a relatively new phenomenon in the Dutch maternity care system. Founded on the notion that pregnancy, birth and puerperium are primarily physiological processes, this system traditionally includes primary as well as secondary (and tertiary) health care. Most pregnant women are healthy (‘low risk’) and therefore start antenatal care with a community midwife [6]. Women with uncomplicated pregnancies can choose where they want to give birth, either at home, in a hospital or in a birth centre. Birth centres are settings where women with uncomplicated pregnancies can give birth in a homelike environment. When complications arise or threaten the birth or pharmacological pain relief is requested, referral to an obstetric unit in a hospital is necessary [7–9]. Birth centres in the Netherlands can be located according to their position in relationship to an obstetric ward, that is, either freestanding, alongside or on-site [8]. For freestanding birth centres, in the case that a woman or baby is referred for obstetric or paediatric assistance, transfer is necessary by car or ambulance. For alongside birth centres, transfers are made via bed or wheelchair. In on-site birth centres, transport in case of referral is not necessary as the secondary caregiver (obstetrician or paediatrician) can enter the birthing room.

In the course of the last decade, several birth centres were established for various reasons (e.g. as result of centralization of hospitals or due to a changing trend in women’s choices for planned place-of-birth), resulting in a substantial rise of births taking place in hospital maternity wards [6]. Furthermore, in 2009 a ministerial steering committee published a report suggesting ways for Dutch maternity caregivers to improve the quality of care [5]. The committee was created following the publication of data from Euro-Peristat showing a relatively high perinatal mortality in the Netherlands as compared to other European countries [10]. Although there were questions about the comparability of data from disparate countries, the data caused concern and led some to conclude that the poor outcomes might be related to the division between primary and secondary care in the Dutch system [11–13]. In their report to the Minister of Health, the committee recommended – among other things – an investigation of the use of birth centres to improve perinatal outcomes, based on an assumption that birth centres might provide higher quality care because they offer a better opportunity for more integrated care. At the time the committee made this recommendation, there was no evidence for that assumption. There were no studies of the nature and degree of integration of birth centres in the Netherlands and there were no data on the effects of integration on quality of care.

The Dutch Birth Centre Study was designed to evaluate the performance of birth centres and their possible added value to the quality of the Dutch maternity care [6]. Because the number of births in most birth centres is very small and the number varies greatly between centres, a necessary first step in the study was to find a reliable way to classify the centres based on common characteristics [14–16]. Besides location, we considered that this classification should be based on characteristics of integration of care given the assumption that birth centres offer an opportunity for more integrated care. Therefore, the aim of this study was to classify birth centres in clusters based on integration characteristics.

## Methods

### Study design

In this study, we used a combination of surveys and qualitative interviews. The data generated by the interviews were used to validate the information from the questionnaires. The study was conducted from January 2014 until August 2015 as part of the Dutch Birth Centre Study [6].

### Theoretical background

**Fig. 1:**
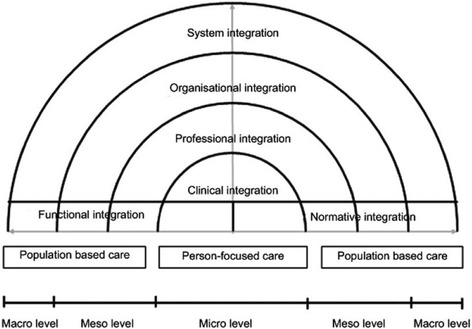
Rainbow Model of Integrated Care. Source: Valentijn et al. (2013)

In this study, we used the concept of integrated care to construct a typology of birth centres. We based our work on a conceptual framework developed by Valentijn et al. [17]. Their “Rainbow Model of Integrated care” (Fig. 1, Table 1) combines the functions of primary care with dimensions of integrated care. The model distinguishes four dimensions that play inter-connected roles on the micro- (clinical integration), meso- (professional and organizational integration) and macro-level (system integration) of a health care system. The model also includes two dimensions that enable the connectivity between the various integration levels (functional and normative integration). The model is specified in a taxonomy consisting of 59 integration determinants, based on literature study and a Delphi study among Dutch experts [3]. In the present study, we used this taxonomy to construct a typology of birth centres in the Netherlands.

**Table 1:** Description of Rainbow model of integrated care

*Clinical* integration refers to the coherence in the primary process of care delivery to individuals. It requires a person-focused perspective and includes the aspect of the clients as co-creators in the care process and shared responsibility between professional and client.
*Professional* integration refers to partnerships between professionals both within and between organisations. These partnerships can be characterised as forms of horizontal and/or vertical integration. To deliver a comprehensive continuum of care, professionals have to share accountability, problem solving and decision-making. As a consequence of this process the professional autonomy is affected and the traditional hierarchy and defined roles become diffused.
*Organisational* integration refers to the extent that services are delivered in a linked-up way. It is necessary to deliver population-based care because of the collective responsibility for the health and wellbeing of a population. Population-based care can be achieved through hierarchical governance structures, network-like governance mechanisms or through marked based governance structures between organisations. In the field of primary care, organisational integration is often realised in a network construction. These complex network arrangements require effective mechanisms of governance and accountability.
*System* integration refers to integration of a health system to realise a holistic approach. It requires a tailor-made combination of structures and processes to fit the needs of people across the continuum of care. Both horizontal and vertical integration are needed to counteract the fragmentation and should be incorporated to provide coordinated care across the entire care continuum.
*Functional* integration supports clinical, professional, organisational and system integration and includes coordination of key support functions as human resources, strategic planning, information management, financial management and quality improvement. Functional integration is the linking of information, management and financial systems around the primary process of service delivery.
*Normative* integration also achieves connectivity. It can provide a common frame of reference, necessary for providing a continuum of care when various actors are in involved [3].

### Development of the birth centre integration questionnaire

Because a validated questionnaire to examine integrated care in birth centres was lacking, we constructed a questionnaire based on a survey that was used to examine integrated care in primary care organizations [18]. For each dimension of integration, we identified four integration determinants. The inclusion procedure for the determinants was based on the highest median score from a panel assembled for a Delphi study [3] and its applicability in birth (centre) care. For each determinant, we formulated a multiple-choice question. The answer categories correspond with stages of integration and range from one (not integrated) to four (fully integrated) forming a nominal scale, with equal weighting between the answer categories [17]. Statements corresponding to each stage were derived from the primary care questionnaire and birth centre practice [18]. The questionnaire was pilot tested by three community midwives familiar with birth centre care. Some questions/statements were adapted based on their comments. Due to the short duration of our study and the limited number of birth centres, we could not test the questionnaire for validity and reliability. In order to compensate for this, we used qualitative interviews to validate the collected information [19, 20] and sequential data collection. In the first phase, quantitative data were collected and described. In the second phase, semi-structured interviews were used to refine the quantitative results obtained in the first phase. Table 2 shows the dimensions of integration and their integration determinants. A translation of the questionnaire (from Dutch to English) used in this study is provided as Additional file 1.

**Table 2:** Integrated care dimensions and determinants of the Rainbow Model of Integrated care

Level	Dimension	Description dimension	Determinant	Description determinant
Micro	Clinical integration	The coordination of person-focused care in a single process across time, place and discipline.	Case management	Coordination of care for clients’ with a high-risk profile (e.g. identifying risks, developing policies and guidance).
			Continuity	The organization of care is aimed to provide fluid care delivery for an individual client.
			Individual multidisciplinary care plan	Implementation of a multidisciplinary care plan at the individual client level.
			Client participation	Clients are (pro) actively involved in the design, organization and provision of care at the operational level.
Meso	Professional integration	Inter-professional partnerships based on shared competences, roles, responsibilities and accountability to deliver a comprehensive continuum of care to a defined population.	Inter-professional education	Inter-professional education for professionals focused on interdisciplinary collaboration.
			Shared vision between professionals	A shared vision between professionals focused on the content of care.
			Multidisciplinary guidelines and protocols	Multidisciplinary guidelines and protocols are implemented in coherence at the operational level.
			Inter-professional governance	Inter-professional governance focused on openness, integrity and accountability between professionals at the operational level (e.g. joint accountability, appeal on pursued policies and responsibilities).
Meso	Organizational integration	Inter-organizational relationships (e.g. contracting, strategic alliances, knowledge networks, mergers), including common governance mechanisms, to deliver omprehensive services to a defined population.	Interest management	A climate that attempts to bridge the various interests (e.g. social, organizational and personal interests) at the operational, tactical and strategic level.
			Performance management	Collective elaborated performance management between organizations within the collaboration.
			Learning organisations	Collective learning power between the organizations within the collaboration (e.g. joint research and development programs).
			Complaints procedure	
Macro	System integration	A horizontal and vertical integrated system, based on a coherent set of (informal and formal) rules and policies between care providers and external stakeholders for the benefit of people and populations.	Available resources	Available resources in the environment of the collaboration (e.g. usable buildings, (over) capacity, professionals and funding streams).
			Stakeholder management	Engagement of various stakeholders (e.g. municipality, patient organizations and health insurance company).
			Good governance	Creating trust towards external stakeholders (e.g. municipality and health insurance company) due to working method, reputation, management, control and/or supervision.
			Environmental climate	Political, economic and social climate in the environment of the collaboration (e.g. market characteristics, regulatory framework, competition).
Micro, meso, macro	Functional integration	Key support functions and activities (i.e. financial, management and information systems) structured around the primary process of service delivery, to coordinate and support accountability and decision making between organizations and professionals to add overall value to the system.	Information management	Aligned information management systems accessible at operational, tactical and strategic level (e.g. monitoring and benchmarking systems).
			Resource management	Coherent use of resources (e.g. collective real estate and funding).
			Service management	Aligned service management for the client (e.g. collective telephone number, counter assistance and 24-h access)
			Regular feedback of performance indicators	Regular feedback of performance indicators for professionals at the operational level to enable them to improve their performance.
Micro, meso, macro	Normative integration	The development and maintenance of a common frame of reference (i.e. shared mission, vision, values and culture) between organizations, professional groups and individuals.	Reliable behaviour	The extent to which the agreements and promises within the collaboration are fulfilled at operational, tactical and strategic levels.
			Visionary leadership	Leadership based on a personal vision that inspires and mobilizes people.
			Quality features of the informal collaboration	Effectiveness and efficiency of the informal collaboration at the operational, tactical and strategic levels (e.g. group dynamics and attention to the undercurrent).
			Trust	The extent to which those involved in the collaboration at operational, tactical and strategic levels trusts each other.

Adapted with permission from: “Towards a taxonomy for integrated care; a mixed-methods study” *(Valentijn 2015)*

### Data collection procedure

The Dutch Birth Centre Study started with the identification of birth locations regarded as birth centres and the development of a definition for birth centres in the Netherlands [6, 21]. Subsequently, managers of the identified birth locations (nationally, 46 in total) were invited to complete the “Dutch Birth Centre questionnaire”. Based on the definition for birth centres, 23 birth centres were identified at the reference date (September 2013). These centres were included in our study and invited to participate. All the managers gave their permission to visit and conduct interviews at their birth centres. We asked managers of birth centres to select two or three care providers, familiar with the organization of the centre, from different professions working within or with the birth centre to be interviewed. Community midwives and, depending on the local situation, maternity care assistants, clinical midwives, obstetric nurse specialists and gynaecologists were invited to be interviewed. Our aim was to form a multi-disciplinary view of the organization and collaboration in and with birth centres. The first author (IB) contacted all participants to explain the study. Quantitative and qualitative data were collected sequentially [20]. Two weeks before the visits and interviews, the “Birth Centre Integration questionnaire” was sent by e-mail to the manager and the selected professionals of each birth centre. One week later, a reminder was sent to the non-responders. Based on the responses to the “Birth Centres Integration questionnaires” and the “Dutch Birth Centre questionnaire”, a specific topic list for each individual birth centre was made before each visit, in order to structure the interviews. An example of this topic list is provided as Additional file 2.

The aim of the interviews was to obtain an additional qualitative view of the degree and nature of integration in the birth centres, and to validate the collected data from the questionnaires. The first author (IB) visited the birth centres and interviewed all respondents.

With participants’ informed consent, all interviews were audiotaped and transcribed verbatim. These transcriptions were collected per birth centre and coded deductively based on the determinants per dimension of integration [22]. As a member check to validate the qualitatively generated data, a summary was written for each birth centre, containing the most important findings from the visits and interviews and characterizing the birth centre on aspects of integration [23, 24]. All respondents reviewed and agreed with these summaries. Because of the multi-disciplinary characteristic of integrated care [15], the research group decided to interpret the answers with the same specific perspectives in mind, namely the context of integration between primary and secondary care and the integration at the birth centre level. Using these perspectives, together with the transcriptions of the interviews and findings from the visits, the first author (IB) completed an integration questionnaire for each birth centre.

### Data analysis

The analysis presented here is based on the quantitative data from the questionnaires. There are two stages in our analysis: (1) calculating the mean score on integration determinants and dimensions for each birth centre, (2) classifying the birth centres based on these mean scores.

#### Calculating integration scores per birth centre

The questionnaire consists of six dimensions of integration, each divided into four determinants. First, we calculated the mean scores of all respondents per dimension for each birth centre (range 1–4). Then we calculated the mean scores of all respondents, including the first author (IB) for the birth centres on the six dimensions of integration (range 1–4). We also computed a total integration score per birth centre by calculating the mean score of the six dimensions combined (range 1–4).

#### Cluster analysis

A four-step procedure was followed to classify the birth centres into different clusters [18]. We conducted a cluster analysis using the mean scores on the six dimensions of integration. First, the appropriate number of clusters was decided by hierarchical cluster analysis using Ward’s method and the Euclidian Distance. Second, a non-hierarchical analysis (K-means method) was performed to validate and adjust the results of the hierarchical procedures. We also performed this analysis using the initial cluster centroids from Ward’s method as seed points [15]. Third, the stability of the cluster assignment between the hierarchical and non-hierarchical method was assessed using Cohen’s coefficient of agreement [25]. A between-subgroup post-hoc test, using a one-way analysis of variance (ANOVA), was used to examine the differences between the clusters on the integration determinants. Fourth, we used the cluster means for each of the six dimensions of integration and the total integration score to provide a meaningful interpretation of the clusters [26–28]. Based on these cluster means, the research group of the Dutch Birth Centre Study judged the results of the clustering appropriate for a meaningful and understandable interpretation. All data analyses were performed using SPSS version 22 (IBM Statistics).

## Results

Between January 2014 and April 2015, 23 birth centres were visited. During these visits, the first author interviewed 69 (managerial) representatives and professionals working within or with a birth centre (range 2–5 per birth centre). Birth centre integration questionnaires were sent to 73 managers and professionals, 61 completed the questionnaire (response rate of 84%). One birth centre was unable to participate in the interviews, because of their workload. However, one professional completed the questionnaire. A researcher (MHi) who was familiar with this birth centre because of her involvement in another part of the Dutch Birth Centre Study also filled in the questionnaire based on her knowledge of this centre.

### Integration scores per birth centre

Table 3 shows the mean scores for each birth centre on the six dimensions of integration and the total integration score based on the mean scores of respondents, including the questionnaire completed by the first author.

**Table 3:** Mean scores birth centres on integration dimensions

Birth Centre	Clinical Integration	Professional Integration	Organizational Integration	Functional Integration	System Integration	Normative Integration	Total Integration
	Mean scores						
1	2.92	2.75	2.58	2.17	2.33	3.58	2.72
2	2.13	1.63	1.50	1.88	2.00	3.13	2.04
3	2.20	1.85	1.85	1.45	2.48	3.15	2.16
4	2.08	2.50	2.67	1.42	2.07	3.50	2.37
5	2.50	2.00	2.19	1.88	2.50	3.50	2.43
6	2.42	1.67	1.88	1.63	2.17	2.71	2.08
7	2.88	2.33	1.94	2.13	2.50	2.88	2.44
8	2.44	3.38	3.38	2.33	2.73	3.19	2.91
9	3.25	3.69	3.50	3.31	3.15	3.63	3.42
10	2.06	2.19	2.38	1.38	2.20	3.13	2.22
11	2.17	2.17	2.71	2.00	3.20	3.71	2.66
12	2.38	1.81	2.63	1.38	2.17	3.00	2.23
13	2.75	2.17	2.58	1.96	2.60	3.33	2.57
14	2.42	2.08	1.75	1.58	1.80	3.63	2.21
15	2.17	2.25	2.25	1.67	2.00	2.67	2.17
16	3.25	2.58	2.42	2.67	2.27	3.42	2.77
17	2.25	2.75	2.50	1.67	2.20	3.33	2.45
18	2.00	1.38	2.00	1.44	2.65	2.92	2.06
19	3.08	3.61	3.31	3.35	2.65	3.21	3.20
20	2.92	2.38	2.48	2.44	2.50	3.38	2.68
21	2.00	3.00	2.75	2.50	3.00	3.50	2.79
22	2.75	2.69	2.56	2.25	2.05	2.94	2.54
23	2.35	3.54	3.25	3.17	3.43	3.75	3.25

### Cluster analysis

Based on the hierarchical cluster analysis (using Ward’s method) the birth centres were classified into three clusters. This classification showed a good agreement with the non-hierarchical cluster analysis (κ = 0.799, *p* < .001) and with this analysis using the initial cluster centroids from the hierarchical method as seed points (κ = 0.865, *p* < .001). Table 4 shows the mean scores on the integration determinants and dimensions of integration for the clusters. Results of the between-subgroup post-hoc comparisons identified statistically significant differences between the clusters for the perceived degree of clinical integration (F(2,20) = 9.64, *p* = .001), professional integration (F(2,20) = 15.0, *p* <.001), organizational integration (F(2,20) = 16.5, *p* < .001), functional integration (F(2,20) = 25.2, *p* < .001) and system integration (F(2,20) = 23.5, *p <* .001*).* No significant differences were found for the normative dimension (F (2,20) = 3.37, *p* = .55).

**Table 4:** Scores of clusters of birth centres on integration determinants and dimensions of integration

n (%)			Total birth centres				Profile A Mono-disciplinary-orientated birth centres (MOBC)				Profile B Mixed-cluster of birth centres (MIBC)				Profile C Multi-disciplinary-orientated birth centres (MUBC)				Subgroup differences F Test
			23 (100)				10 (43.5)				7 (30.4)				6 (26.1)				* *p* < 0.05
																			** *p* < 0.01
																			*** *p* < 0.001
Dimension	Determinant	Range	M	SD	Min	Max	M	SD	Min	Max	M	SD	Min	Max	M	SD	Min	Max	
Clinical integration (CI)	Case management	1–4	3.08	0.32	2.40	4.00	3.04	0.27	2.40	3.33	3.11	0.40	2.75	4.00	3.13	0.33	2.80	3.75	F(2.20) = 0.17
	Continuity	1–4	2.30	0.49	2.00	3.50	2.00	0.00	2.00	2.00	2.52	0.39	2.00	3.00	2.56	0.74	2.00	3.50	F(2.20) = 4.49*
	Individual multidisciplinary care plan	1–4	2.86	0.82	2.00	4.00	2.22	0.25	2.00	2.67	3.79	0.22	3.50	4.00	2.83	0.86	2.00	4.00	F(2.20) = 21.91***
	Client participation	1–4	1.77	0.44	1.00	2.67	1.58	0.35	1.00	2.00	1.99	0.50	1.00	2.67	1.83	0.42	1.33	2.50	F(2.20) = 2.10
	Total CI	1–4	2.49	0.40	2.00	3.25	2.21	0.15	2.00	2.42	2.85	0.23	2.50	3.25	2.55	0.50	2.00	3.25	F(2.20) = 9.64**
Professional integration (PI)	Inter-professional education	1–4	2.05	0.88	1.00	3.75	1.78	0.79	1.00	3.67	1.80	0.55	1.00	2.67	2.81	0.98	1.00	3.75	F(2.20) = 3.77*
	Shared vision between professionals	1–4	2.59	1.03	1.00	4.00	1.87	0.72	1.00	3.33	2.73	0.98	1.67	4.00	3.65	0.32	3.25	4.00	F(2.20) = 11.06**
	Multidisciplinary guidelines and protocols	1–4	2.76	0.73	1.50	4.00	2.41	0.63	1.50	3.33	2.68	0.55	1.50	3.00	3.46	0.64	2.33	4.00	F(2.20) = 5.70*
	Inter-professional governance	1–4	2.46	0.75	1.00	4.00	1.99	0.49	1.00	2.67	2.45	0.34	2.00	3.00	3.25	0.82	2.00	4.00	F(2.20) = 9.50**
	Total PI	1–4	2.45	0.65	1.38	3.69	2.01	0.42	1.38	2.75	2.41	0.28	2.00	2.75	3.23	0.58	2.17	3.69	F(2.20) = 15.01***
Organizational integration (OI)	Interest management	1–4	2.85	0.42	2.00	3.67	2.65	0.48	2.00	3.67	2.81	0.24	2.50	3.00	3.24	0.16	3.00	3.50	F(2.20) = 5.16*
	Performance management	1–4	2.65	1.01	1.00	4.00	1.97	0.81	1.00	3.33	2.65	0.78	1.67	3.67	3.79	0.33	3.33	4.00	F(2.20) = 12.26***
	Learning organisations	1–4	2.86	0.71	1.80	4.00	2.53	0.57	1.80	3.33	2.69	0.51	2.00	3.33	3.60	0.65	2.33	4.00	F(2.20) = 6.89**
	Complaints procedure	1–4	1.65	0.61	1.00	3.25	1.41	0.44	1.00	2.25	1.42	0.47	1.00	2.25	2.31	0.57	1.67	3.25	F(2.20) = 7.50**
	Total OI	1–4	2.48	0.53	1.50	3.50	2.14	0.40	1.50	2.67	2.39	0.24	1.94	2.58	3.15	0.34	2.71	3.50	F(2.20) = 16.46***
System integration (SI)	Available resources	1–4	2.72	0.61	1.17	4.00	2.32	0.47	1.17	2.83	2.70	0.35	2.25	3.33	3.40	0.45	2.75	4.00	F(2.20) = 11.84***
	Stakeholder management	1–4	2.20	0.81	1.00	4.00	1.66	0.47	1.00	2.25	2.10	0.58	1.33	3.00	3.22	0.49	2.67	4.00	F(2.20) = 17.89***
	Good governance	1–4	2.71	0.55	2.00	4.00	2.38	0.25	2.00	2.67	2.67	0.40	2.00	3.00	3.31	0.60	2.50	4.00	F(2.20) = 9.67**
	Environmental climate	1–4	2.03	0.77	1.00	3.25	2.18	0.73	1.00	3.20	1.80	0.76	1.00	3.00	2.06	0.91	1.00	3.25	F(2.20) = 0.48
	Total SI	1–4	2.07	0.61	1.38	3.35	1.55	0.16	1.38	1.88	2.21	0.27	1.88	2.67	2.78	0.57	2.00	3.35	F(2.20) = 25.19***
Functional integration (FI)	Information management	1–4	1.74	0.51	1.00	3.00	1.43	0.23	1.00	1.67	1.86	0.48	1.33	2.67	2.11	0.62	1.50	3.00	F(2.20) = 5.07*
	Resource management	1–4	2.31	1.07	1.00	4.00	1.65	0.60	1.00	2.50	2.68	1.03	1.00	4.00	3.00	1.22	1.00	4.00	F(2.20) = 4.84*
	Service management	1–4	2.39	0.75	1.00	3.75	1.77	0.44	1.00	2.50	2.57	0.53	2.00	3.00	3.22	0.37	2.75	3.75	F(2.20) = 20.06***
	Regular feedback of performance indicators	1–4	1.77	0.86	1.00	4.00	1.35	0.58	1.00	2.50	1.74	0.56	1.00	2.50	2.53	1.10	1.00	4.00	F(2.20) = 4.72*
	Total FI	1–4	2.46	0.43	1.80	3.43	2.17	0.24	1.80	2.65	2.39	0.19	2.05	2.60	3.03	0.30	2.65	3.43	F(2.20) = 23.49***
Normative integration (NI)	Reliable behaviour	1–4	3.52	0.40	3.00	4.00	3.55	0.42	3.00	4.00	3.43	0.46	3.00	4.00	3.60	0.33	3.25	4.00	F(2.20) = 0.30
	Visionary leadership	1–4	3.27	0.55	2.00	4.00	3.01	0.59	2.00	3.67	3.35	0.53	2.50	4.00	3.63	0.21	3.50	4.00	F(2.20) = 2.95
	Quality features of the informal collaboration	1–4	2.80	0.56	1.67	3.50	2.65	0.71	1.67	3.50	2.82	0.47	2.00	3.33	3.01	0.33	2.50	3.33	F(2.20) = 0.79
	Trust	1–4	3.48	0.39	2.67	4.00	3.26	0.34	2.67	3.67	3.56	0.37	3.00	4.00	3.75	0.32	3.25	4.00	F(2.20) = 4.06*
	Total NI	1–4	3.27	0.31	2.67	3.75	3.12	0.31	2.67	3.63	3.29	0.27	2.88	3.58	3.50	0.25	3.19	3.75	F(2.20) = 3.37
Total Integration	Total	1–4	2.54	0.39	2.04	3.42	2.20	0.13	2.04	2.45	2.59	0.13	2.43	2.77	3.04	0.30	2.66	3.42	F(2.20) = 38.0 ***

### Characteristics of clusters of birth centres

**Fig. 2:**
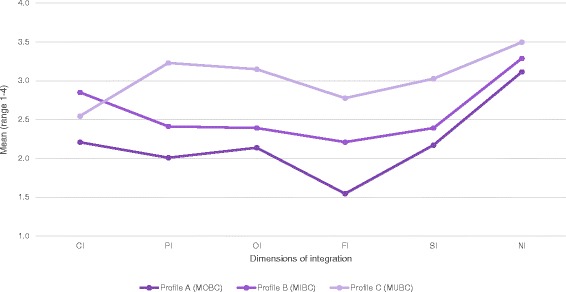
Integration profiles of clusters of birth centres. CI: Clinical Integration, PI: Professional Integration, OI: Organizational Integration, FI: Functional Integration, SI; System Integration, NI: Normative Integration

**Fig. 3:**
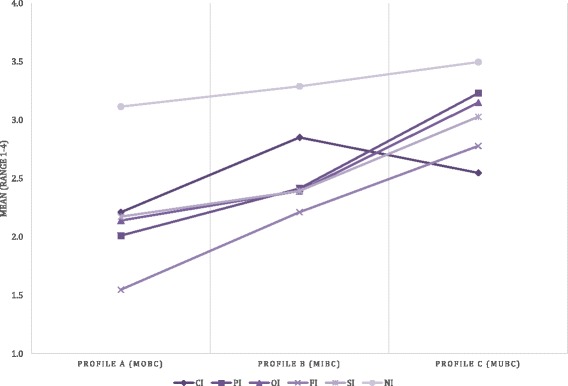
Mean scores dimensions of integration per cluster of birth centres. CI: Clinical Integration, PI: Professional Integration, OI: Organizational Integration, FI: Functional Integration, SI; System Integration, NI: Normative Integration

We labelled the three clusters according to their average characteristics regarding their integration profiles: Mono-disciplinary-oriented birth centres (MOBC)*,* Mixed cluster of birth centres (MIBC) and Multi-disciplinary-oriented birth centres (MUBC) (see Figs. 2 and 3).

#### Profile a: Mono-disciplinary-oriented birth centres (MOBC)

This cluster includes 43.5% of the birth centres (*n* = 10) and is characterized by integration scores on the six dimensions of integration lower than the average score of all birth centres combined and lower than the birth centres in the other clusters. The results of the interviews indicated that birth centres in this cluster are mainly mono-disciplinary (primary care) oriented and are more focused on being a facility to give birth than on improving collaboration between care providers or realizing care integration. They were established as physical facilities to provide an alternative birthplace for low risk births. Some of these birth centres were established to reduce the pressure on hospital maternity wards, others to provide an alternative to home birth. Centres in this cluster are almost all owned by primary care organizations (community midwives or maternity care assistance organizations). They were *not* established with the intention to realize integrated care; their focus is more on practical issues. Protocols, guidelines and other agreements are not discussed in the birth centre itself, but at a different level, at local networks called “local maternity care consultation and cooperation groups” (MCCC-groups) [24]. These networks are located around hospitals.

#### Profile B: Mixed cluster of birth centres (MIBC)

Birth centres in this cluster make up 30.4% (*n* = 7) of the centres. Compared to all birth centres, these centres are characterized by lower scores than average on the professional, organizational and system integration dimensions and relatively higher than average scores on the clinical and functional integration dimensions. Their scores on normative integration were average. Birth centres in this cluster had higher scores on all integration dimensions compared to the cluster MOBC. Compared to cluster MUBC (described below) these centres had higher clinical integration scores and lower scores on the other dimensions. Both the results of the interviews and the questionnaire indicated that birth centres in this cluster differ more from each other in their organization than the centres in the other clusters. It is difficult to indicate an overall characteristic for the birth centres in this cluster except that they have the highest integration scores of all clusters on the clinical dimension (although the differences are not significant when compared to cluster MUBC *(p* = .082*)*)*.* In governance structure and ownership, they are more comparable to the birth centres in cluster MOBC, but on the functional dimension they are more comparable to the birth centres in cluster MUBC.

#### Profile C: Multi-disciplinary-oriented birth centres (MUBC)

Birth centres in this cluster comprise 26.1% (*n* = 6) of the centres and are characterized by integration scores on the six dimensions of integration that are higher than the average scores of all the birth centres. The interviews taught us that birth centres in this cluster are mainly multi-disciplinary (both primary and secondary care) oriented. These birth centres can be regarded as facilities to give birth, with a focus on integrated (birth) care. They have governance structures consisting of both primary and secondary care organizations. The disciplines involved have formulated a joint vision on birth care and the birth centres themselves decide on agreements, protocols, and guidelines.

## Discussion

This study has successfully classified birth centres into three clusters with distinctive characteristics according to their integration profiles, based on the Rainbow model of integrated care and the corresponding taxonomy. Birth centres with similar characteristics were identified in two of these clusters. The third cluster is a mixed cluster of birth centres. We observed statistically significant differences between the clusters. The integration profiles of the clusters show patterns similar to theories about development of collaborative groups (see Figure 3). Birth centres in the MUBC’s show highest scores on the normative dimension, followed by professional, organizational, system, and functional integration. Lowest scores are shown for clinical integration. Integration is to a large extent based on professional behaviour and attitude. Informal coordination mechanisms based on culture, shared values, and vision are essential conditions to make steps towards integration on a professional and organizational level. Functional integration is an important enabler in this process [1, 3]. Patient centred care (clinical integration) is a key concept of integrated care but demands a change in focus in organizations that are traditionally more physician-centred [29].

Our classification system serves to highlight developments in birth care in the Netherlands. As a result of the public and political debate after the publication of the Euro-Peristat studies [10], the Dutch government promoted integration of primary and secondary care as a way to improve perinatal outcomes. In most regions, this collaboration had already existed for many years in in the form of MCCC-groups, but the intensity of the collaboration varied across regions. For birth centres established in regions with a high intensity of collaboration, it seems to be a logical step to arrange more multidisciplinary-orientated centres. Professionals working in these regions seem to be more likely to abandon former structures and to adopt new governing structures (such as shared ownership). These birth centres are found in the MUBC cluster. They are all multi-disciplinary oriented and consider birth centres to be a way to arrange integrated care.

In regions where collaboration between primary and secondary care is less intense, the change towards delivering more integrated care seems to be more difficult. In these regions the distance between primary and secondary care is larger, often based on visionary differences on birth care. Birth centres established in these regions, mostly opt for separate governance structures. These centres focus more on being a comfortable facility to give birth than on improving collaboration between care providers. Some of these centres were established as a result of the centralization of hospitals, offering an alternative to home birth in regions where maternity units in hospitals are too far away. Others were established in competition with neighbouring hospitals, offering a more home-like environment than the current maternity wards, or as a result of too much pressure on hospital maternity wards because of the shift from home to hospital birth [6]. These birth centres are found in the MOBC cluster. Some birth centres in this cluster are located in regions with good collaboration in MCCC-groups, according to the professionals we interviewed. However, they explicitly choose to establish their birth centre separate from clinical care facilities for several reasons, including a desire to keep a separation between physiological and obstetric care and to prevent the demands of clinical care providers from influencing their professional work. Their organization is more fragmented, as shown in the differences on the professional, organizational, system and functional integration dimensions. However, some professionals working within or with birth centres in this cluster stated that the establishment of their birth centre worked to accelerate improved collaboration in their MCCC-group.

Birth centres in the MIBC cluster appear to be in the middle of this process: they are either on their way to more integrated care, but still in separate organizations, or disengaging from a collaboration that may have been too close. Most of the centres in this cluster have existed for a relatively long time (over 5 years) and in our interviews professionals working in or with these centres pointed out that collaboration in their region worked well. However, they have chosen to organize their birth centre apart from secondary care. In addition, they are focussed on achieving integration in the clinical dimension, which is probably closest to their own professional work.

Our classification of birth centres is comparable with observations in other integrated care organizations. Shortell et al. developed a taxonomy of Accountable Care Organizations in the USA, based on eight attributes of these organizations such as size, scope of services offered, and the use of performance accountability mechanisms [14]. They identified three clusters: 1) smaller physician-led practices, which are centred in primary care with a relatively high degree of physician performance management; 2) larger integrated systems, which offer a broad scope of services; 3) hybrid Accountable Care Organizations: moderately sized, joint hospital-physician and coalition-led groups, that offer a moderately broad scope of services. If we overlay our findings on theirs, our MOBCs are like their physician-led practices, MUBCs match up with the integrated system group, and our MIBCs are similar to their hybrid Accountable Care group. Afrite and Mousquès developed a typology of multidisciplinary group practices, health care networks and health care centres in France [16]. They identified five clusters: 1) associative health care centres: relatively old, with frequent multi-professional cooperation and coordination; 2) older municipal health care centres: with a range of non-physicians roles and functions that are more developed than in associative health care centres; 3) recently established but less well integrated health care networks; 4) fairly recent and poorly integrated multidisciplinary group practices; 5) relatively recent and better integrated multidisciplinary group practices. Interpreting their clusters, our MOBC and MUBC belong in their classification of multidisciplinary group practices, health care network and health care centres. The authors also identified different stages of integrated care in the multidisciplinary group practices and a group with more managerial government structures in the health care centres. Valentijn et al. developed a typology of Integrated Care Projects in the Netherlands, based on perceived degree of integration of stakeholders at the professional, organizational and system levels [15]. They identified three clusters in those projects: 1) United Integration Perspectives: characterized by above average integration scores on the three dimensions; 2) Disunited Integration Perspectives: characterized by average scores on system and professional integration and relatively low organizational integration scores; 3) Professional-orientated Integration Perspectives: characterized by low system - average organization – and high professional integration scores. Here too, our classification of birth centres overlaps with their classification: the United Integration Perspective group seems to be comparable with our MUBC cluster, the Disunited Integration Perspectives group with our MOBC cluster and the Professional-orientated Integration Perspectives group with our MIBC cluster. However, we also see some differences in these classifications. Our MUBC and MIBC clusters seem to score more evenly over the different dimensions. Valentijn et al. also compared these groups to effectiveness over time and perceived degree of integration (i.e. rated success). Both the Professional-orientated Integration Perspectives and United Integration Perspective groups showed an increase in collaboration processes over time and Disunited Integration Perspectives Integrated Care Projects were characterized by a decrease in collaboration processes over time. They concluded that effectiveness of Integrated Care Projects is improved when all stakeholders (professionals, managers and policymakers) perceive a high degree of integration. This implies that it is possible that MUBCs and MOBCs could be more effective than MIBCs. In the Dutch Birth Centre Study, we did not assess birth centres on their effectiveness over time. We recommend exploring this in a follow up study. Future studies should focus in more detail on how integration in birth (centre) care influences the effectiveness of collaboration processes.

### Strengths and weaknesses of this study

This study, part of the Dutch Birth Centre Study, is the first study to classify birth centres based on integration profiles. All birth centres in the Netherlands participated in this study, which gives a unique overview of the level of integration in birth centres. For our study, we used a self-constructed questionnaire because a validated was lacking. Using a non-validated questionnaire introduced some problems in the reliability of the results of our study. To minimize these problems, we developed a study design that used a standardised questionnaire combined with personal interviews. All interviews were conducted by the same researcher, who afterwards also completed the questionnaire. In this way, quantitative data derived from a non-validated questionnaire were complemented with qualitative data, increasing reliability. Another possible limitation of this study is the potential bias in the selection and number of respondents per birth centre. We asked managers of birth centres to select two or three care providers from different professions working within or with the birth centre to fill in the questionnaire and to be interviewed, which could result in selection bias. To counter this, the researcher also filled in the questionnaire complementing quantitative data with qualitative data.

### Implications for practice and further research

Our study shows that the birth centre integration questionnaire can differentiate between birth centres based on integration variables. Except on the normative dimension, we identified statistically significant differences between clusters of birth centres on all dimensions. However, the questionnaire needs validation. It is possible that respondents gave socially desirable answers to some integration determinants in the normative dimension (e.g. trust and reliable behaviour). In other dimensions the hierarchy of the answers to some of the questions is questionable. The results of this study and another study (assessing maternity care consultation and cooperation groups) will be used to validate the questionnaire, resulting in a validated instrument for future research in birth care. Based on this validation, the instrument can be used to assess integration aspects in other organizational forms in birth care, both national and international. With this assessment, recommendations for the organization of birth care in the future can be made. It will enable policy makers, health care financiers, professionals and users of maternity care to make an informed choice about the effectiveness of different ways of organizing care at birth.

## Conclusion

Based on the “birth centre integration questionnaire”, birth centres in the Netherlands can be classified in three clusters according to different integration profiles. Although based on a non-validated questionnaire, which has its limitations, results of this study will allow future assessments of the relationship between integration profile and quality, costs, experiences of clients and professionals, and perinatal and maternal outcomes of birth centre care. With this assessment, recommendations for the organization of birth care in the future can be made. Further research is needed to assess the validity of the birth centre integration questionnaire. Based on this validation, the instrument can be used to assess integration aspects in other organizational forms in birth care, both national and international.

## Additional files

Additional file 1: Dutch Birth Centre Integration Questionnaire. A translation of the questionnaire (from Dutch to English) used in this study. (XLSX 18 kb)

Additional file 2: Example of a topic list, used in the interviews. An example of a topic list used in the interviews. (DOCX 20 kb)

## Abbreviations

MCCC-groups: Maternity Care Coordination and Consultation Groups
MIBC: Mixed cluster of birth centres
MOBC: Mono-disciplinary-oriented birth centre
MUBC: Multi-disciplinary-oriented birth centres
WHO: World Health Organization
ZonMw: the Netherlands Organisation for Health Research and Development ZonMw
