# Supplementary material for: Typology of birth centres in the Netherlands using the Rainbow model of integrated care: results of the Dutch Birth Centre Study
Source: BMC Health Serv Res. 2017 Jun 21;17:426. doi: 10.1186/s12913-017-2350-9 (PMC5479044; doi:10.1186/s12913-017-2350-9)
Supplement: Supplementary file 2 — Example of a topic list, used in the interviews. An example of a topic list used in the interviews. (DOCX 20 kb) [file 12913_2017_2350_MOESM2_ESM.docx]

**Example of a topic list used in the interviews**

**Clinical Integration**

1. Case management *<note unclear information form the DBC-IQ >*
2. Individual Multidisciplinary birth plan *<note unclear information form the DBC-IQ >*
3. Client participation *<note unclear information form the DBC-IQ>*
4. Continuity *<note unclear information form the DBC-IQ )>*

*Possible questions:*

1. How does a transfer from the birth centre to the obstetric unit take place? Is everybody doing this on the same way? Are there any differences?
2. How is the birth plan used in practise? Is it used actively? By all disciplines?
3. Is research of client experiences a shared responsibility? How does is take place?
4. How does the birth centre arrange a flowing care process for clients? What actions are taken to arrange this?

**Professional integration**

1. Inter-professional education *<note unclear information form the DBC-IQ >*
2. Multidisciplinary guidelines and protocols *<note unclear information form the DBC-IQ >*
3. Shared vision between professionals *<note unclear information form the DBC-IQ >*
4. Inter-professional governance *<note unclear information form the DBC-IQ >*

*Possible questions:*

1. How is education arranged in the birth centre? Who is participating?
2. Are multidisciplinary protocols written by the birth centre of the Maternity Care Collaboration and Coordination Group? How do professionals work with protocols? What happens when is departed from the protocols? Does the protocols be updated?
3. What is the shared vision? Does everybody feel a shared responsibility in propagate the vision?
4. Does professionals feel a shared responsibility ? Are there any agreements about how share results of care processes

**Organizational Integration**

1. Performance management *<note unclear information form the DBC-IQ >*
2. Learning organizations *<note unclear information form the DBC-IQ >*
3. Complaints procedure *<note unclear information form the DBC-IQ >*
4. Interest management *<note unclear information form the DBC-IQ >*

*Possible questions:*

1. Are targets formulated by the birth centre? What are the targets? How does did work in practice? Is it a shared responsibility to achieve the targets?
2. Is care being evaluated? In what way? By all disciplines together?
3. How does it work when somebody complaints about the care process?
4. Does organizations of professionals discuss interests?

**Functional integration**

1. Service management *<note unclear information form the DBC-IQ >*
2. Information management *<note unclear information form the DBC-IQ >*
3. Regular feedback of performance indicators *<note unclear information form the DBC-IQ >*
4. Resource management *<note unclear information form the DBC-IQ >*

*Possible questions:*

1. Are there any joint activities? Are there information meetings? How are they organized?
2. Do the birth centre have one health record? How is this arranged? Can all professionals involved share the record?
3. Does professionals discuss their data of results of quality indicators?
4. Is there a midwifery practices located in the birth centre?

**System integration**

1. Stakeholder management *<note unclear information form the DBC-IQ >*
2. Available resources *<note unclear information form the DBC-IQ >*
3. Good governance *<note unclear information form the DBC-IQ >*
4. Environmental climate *<note unclear information form the DBC-IQ >*

*Possible questions:*

1. *How does this take place? Joint?*
2. *How do the birth centre handle with insufficient resources?*
3. *How is the birth centre visible for stakeholders? Only the hospital? Shared?*
4. *How does the region is organized? How about competition? The birth centre is established based on completion. How? Are there any problems in collaboration from history? Does competition influence the collaboration?*

**Normative integration**

1. Trust *<note unclear information form the DBC-IQ >*
2. Reliable behaviour *<note unclear information form the DBC-IQ >*
3. Visionary leadership *<note unclear information form the DBC-IQ >*
4. Quality features of the informal collaboration *<note unclear information form the DBC-IQ >*

*Possible questions:*

1. Tell something about trust between professionals working in and within the birth centre. How do you experience this?
2. Do professionals fulfil arrangements? How do you perceive? Shared?
3. What are differences in culture? Between whom?
